# Cholera

**Published:** 1873-07

**Authors:** 


***Cholera.***

The efforts of some of our professional brethren, and of a large portion of the secular press, at the present time, to excite a cholera panic, suggest the propriety of quoting to them the oriental fable of "The Cholera Fiend," who, when accused by the pilgrim in the desert with having slain thirty thousand pilgrims, charged twenty thousand to account of fear, leaving only one-third of the mortality to his own account.

The past history of Asiatic cholera has demonstrated clearly that its progress is regular and its track well defined; it has never yet made it appearance anywhere by concealed approaches, and yet we are told that the disease rages in New Orleans, in Memphis, in Nashville, Louisville, and Cincinnati—developed spontaneously, perhaps. If this be so, it constitutes a "new departure" in the œtiology of the disease, and introduces a new element into its study.

Cases of disease presenting many of the characteristics of cholera occur yearly in most of our large cities, and almost every physician during some "heated term" sees a case which, "if cholera were prevailing, he should be disposed to call cholera."

The morbid influence prevailing just now in the cities south of us, and to some extent in this, seems to be very respectful of persons and places, selecting the filthiest individuals as its victims and the filthiest localities for its field of operation. Heat, unwholesome food, and filth, are doubtless the efficient agents in the generation of the disease now prevailing, and these, neither singly nor combined, will generate cholera. The first factor is not within our control, the others are; and we are sure, that prudence in diet, in avoiding that which is positively unwholesome in food, and temperance in drink, with cleanliness of person and premises, will disarm the other factors of their efficiency as morbific agents. In this city there are numerous foci of disease in the many open privy vaults, left exposed since the fire, and now, under the combined influence of heat and a dry atmosphere, exhaling pestiferous gases far and wide. There are many of these in the heart of the business portion of the city, proclaiming their presence by unmistakable evidence, to even an utter stranger. The authorities cannot occupy themselves more beneficially, in the protection of public health, than to cause the disinfection of these receptacles and the removal of their contents at once. The danger from this source is much more imminent than from Asiatic cholera. W. H.

